# The performance of *Miscanthus* hybrids in saline-alkaline soil

**DOI:** 10.3389/fpls.2022.921824

**Published:** 2022-10-13

**Authors:** Cheng Zheng, Zili Yi, Liang Xiao, Guorong Sun, Meng Li, Shuai Xue, Xiaoying Peng, Meijuan Duan, Zhiyong Chen

**Affiliations:** ^1^College of Agronomy, Hunan Agricultural University, Changsha, China; ^2^College of Bioscience and Biotechnology, Hunan Agricultural University, Changsha, Hunan, China; ^3^Hunan Engineering Laboratory of Miscanthus Ecological Application Technology, Hunan Agricultural University, Changsha, Hunan, China; ^4^Binzhou Polytechnic, Binzhou., Shandong, China

**Keywords:** *Miscanthus*, saline-alkaline soil, agronomic traits, biomass quality, bioenergy

## Abstract

Cultivating the dedicated biomass crop *Miscanthus* on marginal land is a sustainable means of avoiding competition with food crops for arable land. A large proportion of global marginal land is saline–alkaline; however, little is known about the performance of *Miscanthus* in saline-alkaline soil. In this study, *Miscanthus* × *giganteus* and ten other *Miscanthus* hybrids grown in the Yellow River Delta were exposed to low and saline–alkaline soils during the 2016–2018 growing season to evaluate the agronomic traits, biomass quality and the potential productive index of eleven *Miscanthus* genotypes. Plant biomass, plant height, and tiller number significantly decreased in high saline–alkaline soil. In particular, the average plant biomass of ten *Miscanthus* hybrids in low saline–alkaline soil in 2017 and 2018 were 0.21 and 2.25 kg per plant, respectively, and in high saline-alkaline soil were 0.13 and 0.65 kg per plant, respectively. Cell wall, cellulose, and nitrogen content of all genotypes significantly decreased in high saline–alkaline soil, while hemicellulose, ash, sodium, potassium, magnesium, and calcium content significantly increased. However, high saline–alkaline soil had no observable impact on lignin content of *Miscanthus* biomass. The effect of high saline-alkaline on biomass quality parameters could provide important information for the application of *Miscanthus* biomass in saline-alkaline soil. The selected genotypes (A5) could be considered as breeding materials in saline-alkaline soil.

## Introduction

*Miscanthus* is a perennial rhizomatous giant C4 grass currently being developed for the production of lignocellulosic biomass as a fossil fuel replacement and as an eco-industrial crop (Acharya et al., 2018; Zhang et al., 2020; Sen and Baidurah, 2021). The cultivation of *Miscanthus* on marginal land for biomass production would contribute to food security and the efficient use of land resources (Clifton-Brown and Lewandowski, 2002; Tang et al., 2010; Xue et al., 2016; Wagner et al., 2019). Saline–alkaline stress is one of the most common abiotic stresses affecting crops in diverse geographical locations, and the incidence of soil salinity is increasing worldwide (Dendooven et al., 2010). In China, the potential area of saline–alkaline soil planted *Miscanthus* is approximately 2.49 million hectare (Xue et al., 2016). Recent studies show that *Miscanthus* can improve saline–alkaline soil conditions and biodiversity (Pidlisnyuk et al., 2014; Xu et al., 2021). Thus, *Miscanthus* species are considered a non-food crop with a high potential for sustainable production on saline–alkaline soil.

Currently, only one clone, *Miscanthus* × *giganteus* is grown commercially (Xue et al., 2016). However, the capacity of *M.* × *giganteus* to saline-alkaline tolerance still need be improved (Stavridou et al., 2017). In addition, *M.* × *giganteus*, as a triploid infertile clone, cannot be directly established *via* seeds, which hinders its widespread application (Xue et al., 2016). Therefore, the development of new varieties with high saline–alkaline stress tolerance is urgently needed. The two key goals of *Miscanthus* breeding are high plant biomass and superior biomass quality, to meet the requirements of industrial-scale production on saline-alkaline soil. Unlike most crops produced for food, all the above-ground part of *Miscanthus* was harvested, plants grown in saline–alkaline soil are exposed to osmotic stress, ion toxicity, and high pH (Deinlein et al., 2014; Xue et al., 2016). In terms of *Miscanthus*, the plant biomass of *Miscanthus* is significantly reduced at the seedling stage under high salt conditions (Sun et al., 2014; Stavridou et al., 2017). Seventy genotypes of *Miscanthus* were investigated for salt tolerance in the greenhouse, finding that a good alternative for breeding purposes was the diploid species *M. sinensis* (Chen et al., 2017). However, *Miscanthus* as a perennial plant is important to investigate the effect of saline-alkaline on the plant biomass in the field. In addition, genotypes with saline-alkaline tolerance in the field have been developed by interspecific crossing (Zheng et al., 2021). However, whether these genotypes can adapt to higher saline-alkaline soil remains unknown. Moreover, the effect of high saline-alkaline soil on agronomic traits (plant biomass, plant height, stem diameter, tiller number) need be investigated.

Saline–alkaline conditions negatively impact not only plant biomass, but also cell wall biosynthesis (Oliveira et al., 2020) and composition (Birgit and Hartmut, 2000). The main component of *Miscanthus* biomass is the cell wall, which impacts the degradation and transformation of its biomass. For example, high cellulose and hemicellulose contents of *Miscanthus* enhance the yield of fermentative sugars, which are used for bioethanol production (Sekar et al., 2016). The efficiency of converting cell wall polysaccharides into fermentative sugars is determined by the content of lignin and the extent of cross-linking within lignin polymers (Gong et al., 2011; Jagtap et al., 2013; Li et al., 2014). In addition, the contents of ash and ash elements negatively affect power generation (Fu et al., 2014; Masto et al., 2015). Previous studies showed that drought and cold stresses after cell wall composition in *Miscanthus* by decreasing cellulose and substantially increasing hemicellulose, while having little effect on lignin content (Domon et al., 2013; Tim et al., 2016a). Recently, a transcriptomic study showed that cell wall- and ion transportation-related genes were differentially expressed under saline–alkaline stress conditions (Wang et al., 2019). In *Miscanthus*, the ash content of plant biomass increased under saline stress at the seedling stage (Stavridou et al., 2017). However, the effect of high saline-alkaline soil on cell wall components and ash content of *Miscanthus* biomass have so far been sparsely investigated. Moreover, combining plant biomass and quality traits in saline-alkaline soil to investigate the performance of *Miscanthus* would help breeder to develop the new varieties adaptability to saline-alkaline soil.

In this study, we therefore analyzed the agronomic traits and biomass quality of 10 high-biomass *Miscanthus* genotypes grown in Yellow River Delta, China, and *Miscanthus* × *giganteus*, a commercial variety (control) (Muylle et al., 2015). The aims of this study were: (i) investigating the effect of high saline-alkaline on agronomic and biomass quality traits (cell wall, cellulose, hemicellulose, lignin, ash and elemental content); and (ii) combining plant biomass and quality traits to assess the adaptability of these genotypes to saline-alkaline soil.

## Materials and methods

### Plant materials

Eleven *Miscanthus* genotypes were used in this study. Ten of these genotypes were *Miscanthus* hybrids (Y2, Y5, Y11, Y14, Y20, Y21, Y37, Y39, Y44, and A5). Y2, Y5, Y11, Y14, Y21, Y37, Y39, and Y44 selected from 216 *Miscanthus* genotypes in the Yellow River Delta in low saline-alkaline soil during the 2014–2016 growing season, by compared with the biomass yield of *M.* × *giganteus* (data no shown). The 216 *Miscanthus* genotypes derived from the open pollinating offspring of *M. sinensis*, *M. lutarioriparius*, and *M. sinensis* × *M. lutarioriparius* (Supplementary Table 1). Thus, Y2, Y5, Y11, Y14, Y21, Y37, Y39, and Y44 derived from different females. A5 with high biomass yield in the Yellow River delta derived from *M. sinensis* (♀, number: B0605) and *M. lutarioriparius* (♂, number: A0107) (Zheng et al., 2021). *M.* × *giganteus*, as a commercial variety, was used as a control, due to its wide adaptability and high biomass yield (Xue et al., 2016). All genotypes used in this study were obtained from the *Miscanthus* germplasm resource nursery in Binzhou, Shandong province, China (37°25′ N, 117°59′ E). Rhizomes were extracted from the soil, and cut into approximately 300-g pieces, each with two or three buds. Individual rhizome pieces were packaged in air-tight bags to prevent water loss during transportation to the experimental site.

### Experimental site and climatic conditions

**FIGURE 1:**
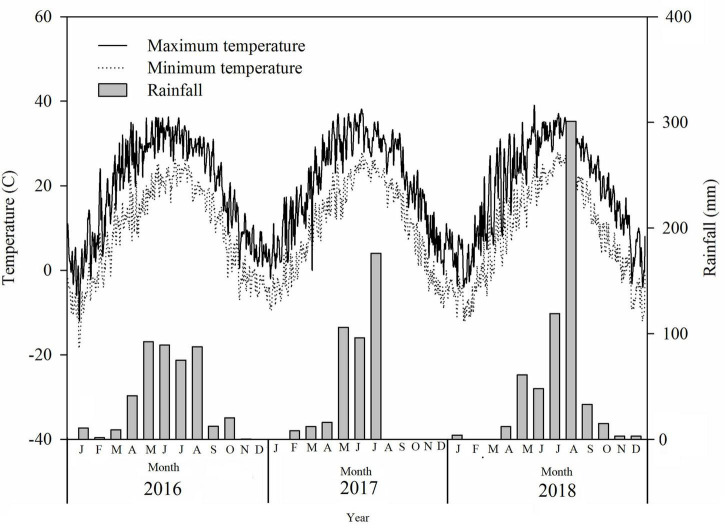
Rainfall and temperature in the experimental sites in 2016, 2017, and 2018.

The experimental site was located in Beihai, Shandong province, China, at the saline–alkaline experimental unit of Hunan Agricultural University (37°38′N, 118°07′E). Two kinds of soil were identified at the experiment site. The physical and chemical properties of these soils are summarized in Table 1. According to the definition of saline–alkaline soil outlined by the United States Department of Agriculture based on the criteria of electrical conductivity (EC; > 4 dS.m^–1^) and pH (> 8.5), soil at the study site was considered as low saline–alkaline under experiment I (EC = 9.04; pH = 2.59). Because the soil EC and pH values in experiment II were higher than those in experiment I, the experiment II soil was defined as high saline–alkaline soil (Table 1). Cotton could be cultivated in experiment I but not in experiment II. The distance between experiment I and II was approximately 300 m. Rainfall and temperature data throughout the experiment were collected by a local meteorological station (Figure 1). Rainfall in 2016, 2017, and 2018 was 439.6, 414.0, and 599.0 mm, respectively.

**TABLE 1:** Physical and chemical features of the soil at the experimental site (the saline–alkaline experimental unit of Hunan Agricultural University, 38°37′N, 118°07′E).

Items	Low saline- alkaline soil (experiment I)	High saline- alkaline soil (experiment II)
pH	9.04	9.62
Electrical conductivity (dS/m)	2.59	12.92
Organic matter (g/kg)	12.12	9.45
Total phosphorus (g/kg)	0.80	0.47
Total nitrogen (g/kg)	2.01	2.02
Total potassium (g/kg)	18.38	24.09
Available phosphorus (mg/kg)	6.10	4.00
Available nitrogen (mg/kg)	48.64	110.89
Available potassium (mg/kg)	338.64	86.61

### Experiment design

Randomized block design was used in both locations, with five rhizomes per genotype in each of the three blocks. Before transplantation, 0.2-m deep furrows were prepared in the field, and rhizomes were placed at the bottom of the furrows, with an inter-rhizome distance of 1 m and row-to-row spacing of 1 m, to obtain a final planting density of 1 rhizome m^–2^. On June 6, 2016, the furrows were filled with soil, and the soil surface was flattened to retain moisture and facilitate soil–rhizome adhesion. To avoid border effects, a supplementary row of A5 rhizomes was planted all the around the experimental site. In addition, to mitigate the edge effect with the neighboring hybrid, ridges were formed between blocks. The areas of location I and location II were both 216 m^2^.

### Management practices

To ensure good root contact with the soil and facilitate seedling emergence, the rhizomes were watered immediately after planting. No irrigation was conducted. In the follow-up experiment. No fertilizer was applied throughout the experiment. Weeds were controlled three times each year by machine hoeing. All plants were survival and overwinter, however, no plant performance data were collected in the first year, because plants of the same genotype showed non-homogenous performance, with only a few tillers per plant.

### Agronomic trait evaluation

Major agronomic traits, including plant biomass, plant height, tiller number and stem diameter, were determined at the end of the second and third growing seasons (on December 8th, 2017 and December 12th, 2018, respectively). The eleven *Miscanthus* genotypes flowered, and stems and leaves of these genotypes were yellow in December. All tillers of each plant were harvested using a Stihl clearing saw (Stihl, Germany) mounted with a steel cutting blade, which was dried to constant weight at 80°C, and plant biomass was weighed. Plant height was measured from the soil surface to the highest point of the last fully expanded leaf. The stem number per plant was measured, as described previously (Zub et al., 2011). To determine the tiller number per plant, only tillers reaching at least 60% of the plant height were counted. To determine the stem diameter, the diameter of a few stems per plant was measured at a height of 5 cm above the soil surface, and the average value was calculated. The above measurements were repeated five times.

### Cellulose, hemicellulose and lignin content measurement

Subsamples were collected from random locations within eleven *Miscanthus* genotype windrows in the low and high saline-alkaline soil after the measured agronomic traits. Gravimetric measurements of neutral detergent fiber (NDF), acid detergent fiber (ADF) and acid detergent lignin (ADL) were determined by using the Van Soest method (Van Soest, 1967). NDF, a measure of cell wall content, is the residue left after refluxing for 1 h in a neutral buffered detergent solution. ADF is a measure of cellulose and lignin, the residue remaining after refluxing the samples in a solution of cetyltrimethylammonium bromide (CTAB) in 2 M sulfuric acid. ADL was measured by treating ADF with 72% sulfuric acid to solubilise the cellulose to determine crude lignin (Allison et al., 2011). The cellulose, hemicellulose and lignin contents of samples were estimated using the following three equations:


(1) Cellulose⁢content=ADF-ADL

(2) Hemicellulose⁢content=NDF-ADF

(3) Lignin⁢content=ADL

### Ash content of plant biomass

The ash content of samples was determined as a percentage of dry matter (%DM) in accordance with the British Standard method (CEN/TS14775: 2004).

### Elemental content of plant biomass

The contents of various elements, including nitrogen (N), phosphorus (P), sodium (Na), potassium (K), calcium (Ca) and magnesium (Mg), in plant biomass were determined. nitrogen analyses were carried out using the Kjeldahl method. Vanadium-molybdenum yellow colorimetry was used to determine the P content. To determine Na, K, Mg, and Ca contents, 0.5 g of dried biomass of each sample was dissolved in 8 ml of HNO_3_ (65%). Then, 4 ml of H_2_O_2_ was added to the reaction to remove color. Samples were then digested in a microwave at 150°C and 24.16 bar for 40 min. The digested samples were filtered through Whatman filter paper and subjected to inductively coupled plasma-optical emission spectrometry (ICP-OES) to determine the content of various elements (Vista Pro; Varian Inc., Palo Alto, CA, USA).

### Statistical analysis

Analysis of variance (ANOVA) was performed to determine the significance of genotype, location, year and interaction. To analyze agronomic and quality traits, genotype and location were set as fixed factors. Agronomic traits were analyzed using Model I, as shown below. Because biomass quality traits were measured only in 2018, these were measured using model II.


(4) $$\mathrm{Model}\,I:Y_{\mathrm{ijkl}}=u+G_{i}+L_{j}+Y_{k}+B_{l}+{\left(\mathrm{GL}\right)}_{\mathrm{ij}}+{\left(G\,Y\right)}_{i\,k}+{\left(L\,Y\right)}_{j\,k}+{\left(G\,L\,Y\right)}_{i\,j\,k}+e_{i\,j\,k\,l}$$

(5) $$\mathrm{Model}\,\mathrm{II}:Y_{\mathrm{ijl}}=u+G_{i}+L_{i}+B_{l}+{\left(\mathrm{GL}\right)}_{\mathrm{ij}}+e_{\mathrm{ijl}}$$

Where Y*_*ijkl*_* and Y*_*ijl*_* are the response variables; *u* is the grand mean; G*_*i*_* is the genotype effect; L*_*j*_* is the location effect; Y*_*k*_* is the year effect; B*_*l*_* is the block effect; (GL)*_*ij*_* represents the genotype × location interaction; (GY)*_*ik*_* represents the genotype × year interaction; (LY)*_*jk*_* represents the location × year interaction; (GLY)*_*ijk*_* represents the genotype × location × year interaction; and e*_*ijkl*_* and e*_*ijl*_* represent the residual error.

To identify significant genotypic differences within low or high saline-alkaline treatment, multiple comparison analyses were performed using Duncan’s multiple range test. Significant differences in agronomic traits and biomass composition between the two groups of genotypes in low and high saline-alkaline soil treatments were evaluated using unpaired two-sample *t*-tests at *P* < 0.05.

In addition, The K^+^/Na^+^ ratio in relation to plant biomass and biomass quality-related variables were conducted by regression analysis (SPSS 19.0, USA).

## Results

### Agronomic traits of *Miscanthus* in low and high saline–alkaline soil

**FIGURE 2:**
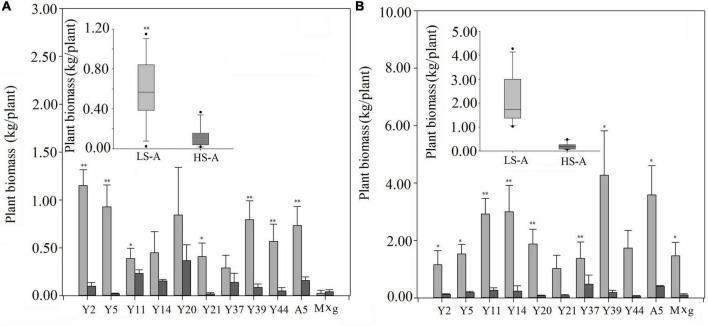
Plant biomass under low saline–alkaline soil (LS-A) (the light bars) and high saline-alkaline soil (HS-A) (the dark bars), of 11 genotypes of *Miscanthus* in 2017 **(A)** and 2018 **(B)**. Significance is denoted by *P < 0.05 and ^**^P < 0.01.

**FIGURE 3:**
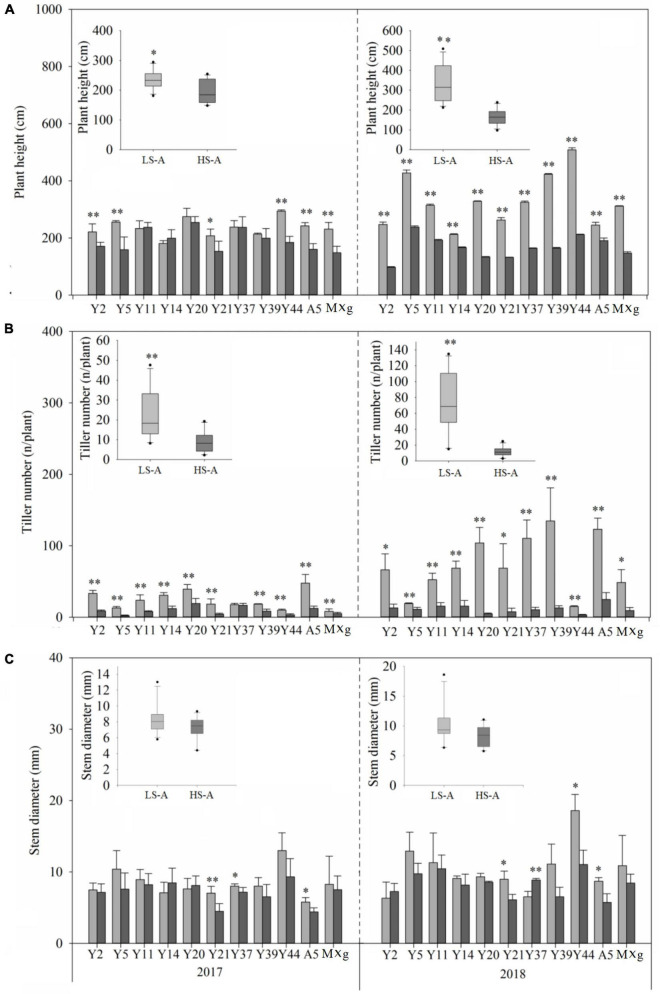
**(A)** Plant height, **(B)** tiller number, and **(C)** stem diameter of 11 genotypes of *Miscanthus* grown in low saline–alkaline soil (LS-A) (the light bars) and high saline–alkaline soil (HS-A) (the dark bars). Significance is denoted by *P < 0.05 and ^**^P < 0.01.

High saline–alkaline stress was found to have a significant (*P* < 0.05) impact on plant biomass, plant height, tiller number and stem diameter (Figures 2, 3 and Table 2). Mean plant biomass in low saline–alkaline soil was 0.60 and 2.19 kg per plant in 2017 and 2018, respectively. On average, high saline–alkaline stress conditions in this experiment significantly (*P* < 0.01) reduced plant biomass by 80% in 2017 (0.12 kg per plant) and 90% in 2018 (0.20 kg per plant). Mean plant height of all genotypes was significantly (*P* < 0.01) reduced by 44 and 160 cm in 2017 and 2018, respectively. Additionally, mean plant height was lower in high saline–alkaline soil than in low saline–alkaline soil in both years (191 vs. 235 cm in 2017; 167 vs. 327 cm in 2018). Similar results were observed for tiller number. Stem diameter was significantly (*P* < 0.01) influenced by high saline–alkaline conditions. Stem diameters of Y21, Y37 and A5 genotypes were significantly (*P* < 0.01) reduced under high saline–alkaline soil conditions. Genotype-location interactions had a significant impact on agronomic traits, expect stem diameter. In 2018, both A5 and Y39 genotypes showed relatively high yields (3.6 and 4.3 kg per plant, respectively) in low saline-alkaline soil; however, in high saline–alkaline stress, the yield of A5 (0.41 kg per plant) was considerably higher than that of Y39 (0.18 kg per plant). In addition, year–location interaction had a significant (*P* < 0.01) impact on plant biomass.

**TABLE 2:** Significance of effect of genotype (G), location (L), year (Y), genotype × location (G × L), genotype × year (G × Y), location × year (L × Y), genotype × location × year (G × L × Y) on plant biomass, plant height, tiller number and stem diameter (model I).

Variance	Plant biomass	Plant height	Tiller number	Stem diameter
G	**	**	**	**
L	**	**	**	**
Y	**	**	**	**
G × L	**	**	**	NS
G × Y	**	**	**	NS
L × Y	**	**	**	**
G × L × Y	**	**	**	NS

Block (B) showed NS, with no shown in table. NS, no significance; **P < 0.01.

### Cell wall, cellulose, hemicellulose and lignin contents of *Miscanthus* genotypes in low and high saline–alkaline soils

**FIGURE 4:**
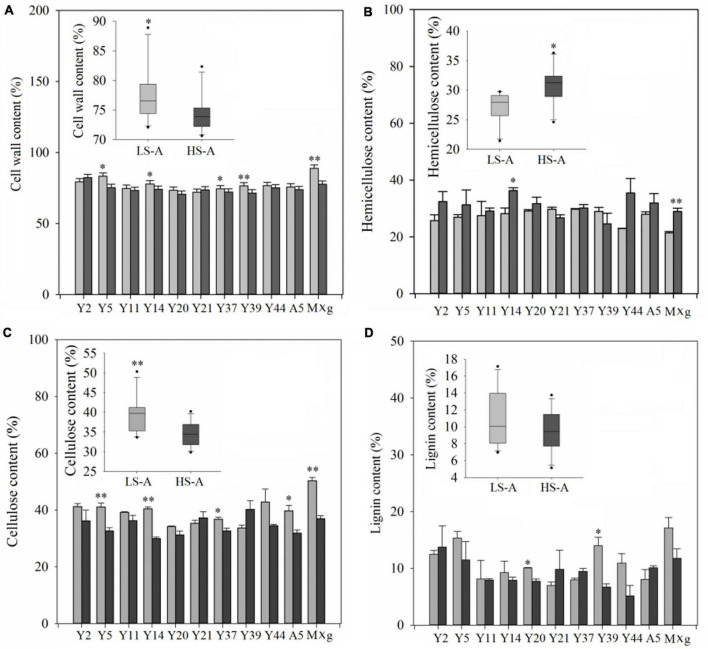
**(A)** Lignocellulose content, **(B)** hemicellulose content, **(C)** cellulose content, and **(D)** lignin content of 11 genotypes of *Miscanthus* grown in low saline–alkaline soil (LS-A) (the light bars) and high saline–alkaline soil (HS-A) (the dark bars). Significance is denoted by *P < 0.05 and ^**^P < 0.01.

High saline–alkaline stress had a significant (*P* < 0.05) effect on most of the biomass quality traits of *Miscanthus* genotypes, including cell wall, cellulose and hemicellulose content (Table 3). Mean cell wall and cellulose contents of all 11 genotypes in high saline–alkaline soil (74.53 and 34.51%, respectively) were lower than those in low saline–alkaline soil (77.56 and 39.54%, respectively), which was a significant difference (*P* < 0.05; 3.03 and 5.03%, respectively) (Figures 4A,C). In particular, the cell wall and cellulose contents of Y5, Y14, Y37 and *M.* × *giganteus* in high saline–alkaline soil were significantly (*P* < 0.05) lower than those in low saline–alkaline soil. In low saline-alkaline, the cell wall of *M.* × *giganteus* was 11. 40% higher than mean cell wall content (88.90 vs. 77.50%). In high saline-alkaline, although the cell wall of *M.* × *giganteus* was 3.13% higher than mean cell wall content (77.66 vs. 74.53%), the cell wall of Y2 was highest (82.33%) (Figures 4A,C). Similarity, the cellulose content of *M.* × *giganteus* was 10.79% higher than mean cellulose content in low saline-alkaline soil (50.33 vs. 39.54%). In high saline-alkaline soil, the cellulose content of Y39 was highest (40.22%). Interestingly, the mean hemicellulose content across all genotypes was significantly decreased by 3.68% in low compared with high saline–alkaline soil (27.10 vs. 30.78%) (Figure 4B). The mean hemicellulose of ten genotypes were 2.04 and 3.23% higher than that *M.* × *giganteus* in high and low saline-alkaline soil, respectively (30.96 vs. 28.92% and 27.66 vs. 21.43%). In addition, the hemicellulose contents of Y14 and *M.* × *giganteus* under high saline–alkaline conditions were significantly (*P* < 0.05) higher than that under low saline–alkaline conditions (Figure 4B). Genotype, high saline–alkaline soil conditions and location–genotype interactions had no observable impact on lignin content (Table 3). However, the mean lignin content of *M.* × *giganteus* in low and high saline-alkaline was the highest (14.40%) (Figure 4D).

**TABLE 3:** Significance of effect of genotype (G), location (L), year (Y), genotype × location (G × L) on quality (model II).

Variance	G	E	G × L
Cell wall	*	*	NS
Hemicellulose	NS	*	NS
Cellulose	NS	*	**
Lignin	NS	NS	NS
Ash	**	**	NS
Nitrogen	*	**	**
Sodium	NS	NS	**
Potassium	NS	NS	**
Magnesium	NS	**	**
Calcium	NS	NS	**
Phosphorus	NS	NS	**

Block (B) showed NS, with no shown in table. NS, no significance; *P < 0.05; **P < 0.01.

### Ash and elemental contents of *Miscanthus* under low and high saline–alkaline conditions

**FIGURE 5:**
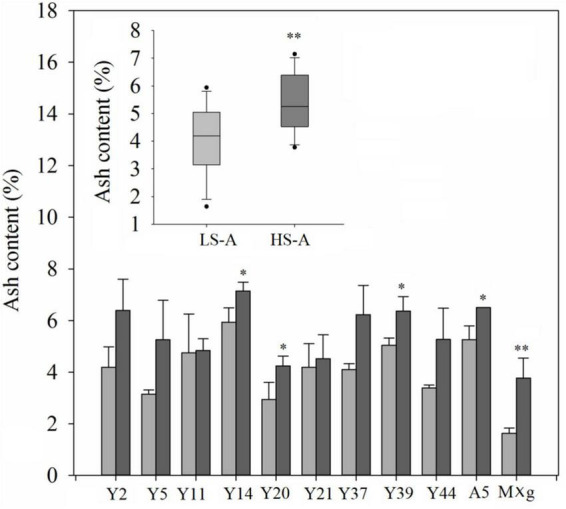
Ash content of 11 genotypes of *Miscanthus* grown in low saline–alkaline soil (LS-A) (the light bars) and high saline-alkaline soil (HS-A) (the dark bars). Significance is denoted by *P < 0.05 and ^**^P < 0.01.

Higher saline–alkaline stress significantly (*P* < 0.01) and consistently increased the ash content of *Miscanthus*, while genotype–location interaction had no significant effect on this trait (Table 3). The mean ash content of all genotypes in high saline–alkaline soil was significantly higher than that in low saline–alkaline soil (5.50 vs. 4.06%); this was particularly evident in the ash content of Y14 (7.14 vs. 5.93%), Y20 (4.25 vs. 2.94%), Y39 (6.37 vs. 5.04%), A5 (6.50 vs. 5.26%) and *M.* × *giganteus* (3.77 vs. 1.64%) (Figure 5).

**FIGURE 6:**
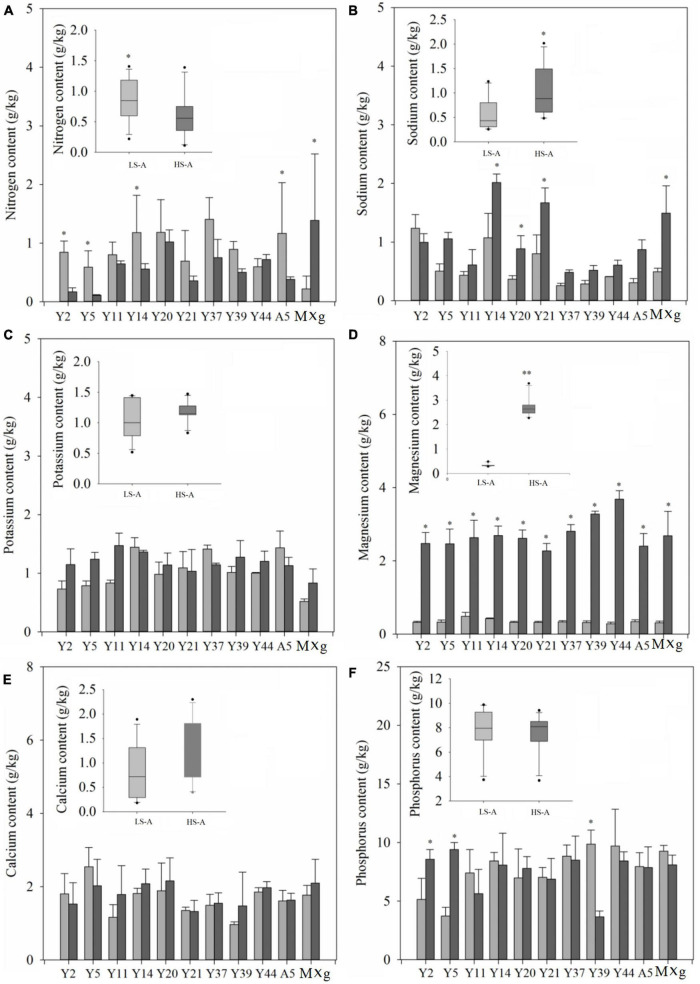
The nitrogen **(A)**, sodium **(B)**, potassium **(C)**, magnesium **(D)**, calcium **(E)**, and phosphorus **(F)** content of 11 genotypes of *Miscanthus* grown in low saline–alkaline soil (LS-A) (the light bars) and high saline–alkaline soil (HS-A) (the dark bars). Significance is denoted by *P < 0.05 and ^**^P < 0.01.

The mean nitrogen (N), phosphorus (P), sodium (Na), potassium (K), calcium (Ca), and magnesium (Mg) contents of all genotypes and each individual genotype in low and high saline-alkaline soil are shown in Figure 6. Saline–alkaline soil level had a significant (*P* < 0.01) impact on nitrogen and magnesium contents (Table 3). The mean nitrogen content across all genotypes was significantly (*P* < 0.05) higher in low saline–alkaline soil than in high saline–alkaline soil (0.82vs. 0.59 g/kg) (Figure 6). Furthermore, except Y44 and *M.* × *giganteus*, all genotypes showed lower nitrogen content in high saline–alkaline soil than in low saline–alkaline soil. By contrast, the sodium and magnesium contents across all genotypes were significantly (*P* < 0.05) elevated in high saline–alkaline soil compared with low saline–alkaline soil. In particular, the average magnesium content across all genotypes in high saline–alkaline soil was more than eight-fold higher than that in low saline–alkaline soil (2.77 vs. 0.33 g/kg). Although the mean potassium, calcium and phosphorus contents across all genotypes were greater in high saline-alkaline soil compared with low saline–alkaline soil, none of these differences were significant.

### K^+^/Na^+^ ratio in relation to yield and quality-related variables

**FIGURE 7:**
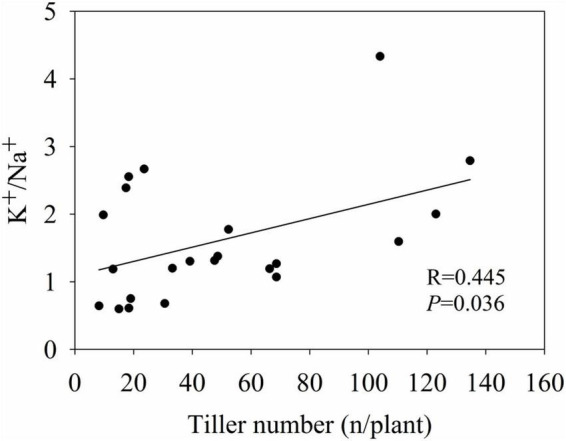
The linear relationship between tiller number and K^+^/Na^+^.

The K^+^/Na^+^ ratio of various genotypes was analyzed in relation to their plant biomass and quality-related variables by linear regression analysis. Among all variables tested, only tiller number showed a significant linear relationship with the K^+^/Na^+^ ratio (*R* = 0.445, *P* = 0.036) (Figure 7).

## Discussion

### High saline–alkaline soil influences agronomic traits

Agronomic traits of *Miscanthus*, including plant biomass, plant height, tiller number and stem diameter, were analyzed in this study to evaluate its potential as a bioenergy crop (Atienza et al., 2003; Liu et al., 2021). Plant biomass, as directly harvested trait, was significantly reduced in high saline–alkaline soil, which was consistent with the results of other bioenergy crops such as switchgrass (Liu et al., 2014; Zhang et al., 2021). The plant biomass of *M.* × *giganteus* was previously found to be reduced by 56% at 5–15 dS m^–1^ EC under high salinity stress (Stavridou et al., 2017). However, in the current study, plant biomass in high saline–alkaline soil (12.92 dS m^–1^) was 10-fold lower than that in low saline–alkaline soil (2.59 dS m^–1^). A possibility could explain that an effect of saline–alkaline stress on *Miscanthus* agronomic traits may be stronger than that of salinity or alkalinity alone. High photosynthesis and efficient water use still maintain biomass yield under alone salinity stress (Yan et al., 2015). However, under alkaline stress, the H^+^ content of apoplast is vital for the maintenance of plasma membrane potential, which regulates cell growth (Yang et al., 2021). High pH value may have an adverse effect on the H^+^ content of apoplast. However, the alkaline stress response of *Miscanthus* remains unknown. The conjecture needs to be confirmed.

In addition, the conclusion of Stavridou report was based on the salt tolerance in the seedling phase, when plant height and stem diameter mainly contributed to plant biomass. Interestingly, we found that tiller number was more sensitive than plant height and stem diameter. Moreover, no significant difference stem diameter between low saline-alkaline soil and high saline-alkaline soil was observed. The reason may be that may be that stem diameter is less affected by the location. Previous study showed that the location had no significant impacts on stem diameter (Jeżowski et al., 2011). However, the investigation of *Miscanthus* in the field is still necessary for breeding new varieties with saline-alkaline tolerance.

### High saline–alkaline soil changes the cell wall compositions and ash content

Cell wall components, including cellulose, hemicellulose and lignin, constitute most of the *Miscanthus* biomass (Schäfer et al., 2018). The cell wall composition of *Miscanthus* varies with environment (Lewandowski et al., 2003; Jensen et al., 2017). In the current study, the cell wall content of *Miscanthus* biomass was reduced by high saline–alkaline stress, whereas the ash content increased, consistent with the findings in switchgrass (Liu et al., 2014). Moreover, results obtained in this study under high salinity stress were similar to those obtained previously (Stavridou et al., 2017). With saline-alkali stress, ash and lignin content of switchgrass biomass increased, and cellulose and hemicelluloses content of switchgrass biomass decreased (Liu and Wu, 2014). In the study, the hemicellulose content of *Miscanthus* was significantly higher in high saline–alkaline than in low saline–alkaline soil, while the lignin content of *Miscanthus* showed no significant difference between low and high saline-alkaline conditions. Under drought stress, hemicellulose content of *Miscanthus* also increased (Tim et al., 2016a), suggesting that adjustment of cell wall components to saline–alkaline and drought stress may be similar. An increase in the relative proportion of hemicellulose content, along with a decrease in the relative proportion of cellulose content, may therefore enable the plant cell walls to uphold their structural rigidity without compromising plasticity under high saline–alkaline conditions (Gall et al., 2015). This may explain the results of the current study. Interestingly, the lignin content of *Miscanthus* showed no significant difference between low and high saline–alkaline conditions, which should be studied in the future study. Although the soil EC and pH between location I and location II were mainly different, other differences (available nutrients) also existed, which may be explain why the cell wall changes observed in this study differed from those in other studies.

The ash content of *Miscanthus* was substantially higher in high saline–alkaline soil than in low saline–alkaline soil; similar results were observed by Stavridou et al. (2017). The ash of biomass combustion has a low melting point and is easy to adhere to the wall of furnace and superheater (Baxter et al., 2012). Furthermore, elements, released from ash in the combustion chamber, can be corrosive and can cause slagging and fouling (Lewandowski and Kicherer, 1997; Tim et al., 2016b). In addition, although we tried to make sure that all environmental factors, except for soil saline-alkaline, were similar. The soil nutrition was still different, which may be have impacts on elemental content of biomass. In the near future study, the effect of the elemental content of soil on elemental content of biomass will be investigated.

### Implications of the current results for breeding saline–alkaline stress tolerant *Miscanthus* varieties

Although optimizing the establishment of *Miscanthus* on saline–alkaline soil may improve its adaptability, breeding new varieties grown on saline–alkaline soil is still a priority (Clifton-Brown et al., 2019; Zheng et al., 2021). Screening for saline–alkaline tolerance in genotypes selected from natural germplasm resources or developed by interspecific hybridization may address this pressing need (Zheng et al., 2021). *Miscanthus* species exhibit wide variation in saline stress tolerance (Chen et al., 2017), and screening for saline–alkaline stress tolerant genotypes from germplasm resources is possible (Zheng et al., 2019). These genotypes could be used directly for breeding new varieties.

Currently, production of bioethanol for *Miscanthus* biomass feedstocks, were initially identified as the most promising value chains (Hessini et al., 2019; Moll et al., 2020). We identified that *Miscanthus* genotypes A5 was significantly superior to *M*. × *giganteus* in low or high saline-alkaline soil, which are therefore promising genotypes for breeding saline–alkaline tolerant varieties. Although the plant biomass of the three genotypes in saline–alkaline soil was higher than that of *M*. × *giganteus*, their biomass quality still needs be improved for the production of bioethanol. In this study, the biomass quality of *M*. × *giganteus* was better than that of the other 10 genotypes assayed, owing to low lignin content contributing to biomass conservation. Therefore, it is important that how to improve biomass quality and reduce lignin content.

Cell wall quality traits is determined by many polygenic traits (Ragauskas et al., 2014; Van der Cruijsen et al., 2021). The highly diverse *Miscanthus* germplasm is an attractive resource for the selection of genotypes with desirable cell wall properties (Yong, 2012; Zhao et al., 2014). In the current study, among various *Miscanthus* species, *M. lutarioriparius* showed relatively better biomass quality, which could be used for the production of bioethanol, with cellulose and hemicellulose contents more than 80%, and lignin content less than 12% (Zheng et al., 2019). In the future, backcross could be used to improve the biomass quality, and the biomass quality traits of *M. lutarioriparius* may be introduced into hybrids with high yielding potential in saline–alkaline soil (Zheng et al., 2022). Meanwhile, high saline-alkaline soil had significant impacts on biomass quality traits, which may be negatively influences biomass conversion. Homogeneous biomass feedstocks help control production parameters. Thus, In the breeding process, the genotype, that biomass quality traits are not sensitive to environment, should be noticed.

In addition, analysis of biomass yield traits such as plant biomass, plant height and tiller number is an effective, but not an efficient, method for evaluating the saline–alkaline stress tolerance of *Miscanthus*, because its agronomically relevant traits can be evaluated in a representative matter only after a growth period of at least 2–3 years (Lewandowski et al., 2016). Thus, this is a time-consuming and laborious process. However, salt tolerance-related molecular markers are not yet available. We found that the tiller number of *Miscanthus* was significantly (*P* < 0.001) influenced by high saline–alkaline soil. Moreover, the K^+^/Na^+^ ratio of aboveground biomass, one of the most important physiological indicators used to estimate plant salt tolerance (Wu et al., 2013; Płażek et al., 2014; Chen et al., 2017), was significantly (*P* = 0.036) correlated with tiller number, implying that a preliminary comparison of yield potential can be made on the basis of K^+^/Na^+^ ratio. In addition, a previous study indicated that several salt tolerant *Miscanthus* species at the seedling stage showed relatively high K^+^/Na^+^ ratios in the shoots under salt stress conditions (Sun et al., 2014; Chen et al., 2017). Therefore, utilization of the K^+^/Na^+^ ratio of aboveground biomass, as an indicator of saline–alkaline stress tolerance, may speed up the breeding of *Miscanthus* genotypes suitable for cultivation in saline–alkaline soil.

## Conclusion

Although *M.* × *giganteus* is a widely used commercial variety, its saline–alkaline tolerance is only moderate. In this study, we demonstrated that the detrimental effects of high saline–alkaline stress on plant biomass, plant height and tiller number are significant. Owing to its high saline–alkaline tolerance and biomass quality, the plant biomass of A5 was significantly higher than that of *M*. × *giganteus* in 2018, under low and high saline-alkaline, which are therefore promising genotypes for breeding saline–alkaline tolerant varieties. However, the biomass quality of A5 still need be improved, example for high ash content. In future studies, *Miscanthus* genotypes should be propagated to investigate and confirm plant biomass improvements in multiple saline–alkaline environments. Moreover, we found that the effects of high saline–alkaline soil on *Miscanthus* biomass quality traits were significant, with decreases in cell wall, cellulose, and nitrogen content, and increases in hemicellulose, ash, sodium, potassium, magnesium, and calcium content. No significant difference in lignin content of *Miscanthus* grown in low and high saline-alkaline soil was observed. These results provide a basis for optimizing the industrial utility of *Miscanthus* biomass. However, the effects of saline–alkaline soil on saccharification potential of *Miscanthus* biomass remains poorly investigated. Thus, in further studies, the impact of saline–alkaline soil on industrial production of *Miscanthus* should be investigated.

## Data availability statement

The original contributions presented in this study are included in the article/Supplementary material, further inquiries can be directed to the corresponding authors.

## Author contributions

CZ and ZY conceived and designed the experiments. CZ analyzed the data and wrote the manuscript. LX, GS, ML, SX, and XP conducted the experiments. MD and ZC executed the projects and provided the funding. All authors contributed to the article and approved the submitted version.
