# Supplementary material for: The performance of Miscanthus hybrids in saline-alkaline soil
Source: Front Plant Sci. 2022 Oct 13;13:921824. doi: 10.3389/fpls.2022.921824 (PMC9608507; doi:10.3389/fpls.2022.921824)
Supplement: Supplementary file 1 [file Table_1.docx]

**Supplementary Table 1.** Individual plants were selected from the offspring of female parent.

| species | Female parent number | Origin | The number of alternative individuals individuals |
| --- | --- | --- | --- |
|  | A0106 | Tonglin, Anhui | 5 |
|  | A0110 | Maanshang,Anhui | 5 |
|  | A0111 | Changde,Hunan | 12 |
|  | A0112 | Huarong, Hunan | 11 |
| *M.lutarioriparius* | A0118 | Changde,Hunan | 10 |
|  | A0119 | Yuanjiang,Hunan | 12 |
|  | C0110 | Hanshou, Hunan | 7 |
|  | C0111 | Jingxian, Jiangxi | 8 |
|  | C0121 | Dongzhi, Anhui | 13 |
|  | C0131 | Fangchang, Anhui | 14 |
|  | C0119 | Wuzhishan, Hainan | 1 |
|  | A0433 | Shaoyan, Hunan | 2 |
|  | B0341 | Nandang, Guangxi | 2 |
|  | B0507 | Huaihua, Hunan | 4 |
|  | B0535 | Jinyun, Chongqing | 3 |
|  | B0632 | Changsha, Hunan | 8 |
| *M. sinensis* | B0636 | Yizhang, Hunan | 8 |
|  | C0424 | Jingganshan, Jiangxi | 7 |
|  | C0503 | Quanzhou, Guanxi | 1 |
|  | C0634 | Chongqing | 5 |
|  | D0123 | Yichong, Jiangxi | 5 |
|  | VI-A-2 | - | 2 |
|  | VI-A-5 | - | 6 |
|  | VI-B-1 | - | 5 |
|  | VI-B-15 | - | 10 |
|  | VI-B-18 | - | 6 |
|  | VI-B-22 | - | 6 |
| 1. *sinensis*×*M.lutarioriparius* | VI-B-24 | - | 1 |
|  | VI-B-3 | - | 6 |
|  | VI-C-12 | - | 3 |
|  | VI-C-22 | - | 11 |
|  | VI-C-24 | - | 4 |
|  | VI-C-9 | - | 3 |
|  | VII-B-15 | - | 1 |
|  | XZM3 | - | 9 |
| Total |  |  | 216 |
